# Composition and size of close social networks among older adults in same-sex and different-sex couples

**DOI:** 10.1093/geronb/gbag185

**Published:** 2026-09-10

**Authors:** Christopher A Julian

**Affiliations:** LGBTQ+ Policy Lab, Vanderbilt University, Nashville, Tennessee, United States; (Social Sciences Section)

**Keywords:** Chosen family, LGBTQ+, Friendship, Queering

## Abstract

**Objectives:**

This study examines differences in the composition and size of the close social network among older adults in same-sex and different-sex coresidential unions.

**Methods:**

Using pooled, probability-based, cross-sectional data from the 2006–2022 Health and Retirement Study, weighted fractional probit and negative binomial regression models were estimated to examine differences in close network composition (proportions of close children, immediate family members, and friends) and network size (number of close ties).

**Results:**

Compared with older adults in different-sex unions, those in same-sex unions reported a higher proportion of friends and a lower proportion of children and immediate family members in their close network, though overall network size was comparable. Supplemental analyses suggested heterogeneity in these patterns. The same-sex versus different-sex gap in friend composition was larger among males than females and was similarly larger among those who were cohabiting than married. Finally, in terms of network size, males in same-sex couples reported smaller close networks than males in different-sex couples, whereas network size was equivalent between females in same-sex and different-sex couples.

**Discussion:**

Beyond their partners, older adults in same-sex unions cultivate distinctly friend-centered close networks. This pattern reflects a history of intentionally built chosen ties that queers dominant assumptions about who partnered older adults designate as close. These findings affirm the importance of policies and practices that acknowledge diverse family arrangements, particularly those rooted in chosen kinship, as a fundamental step toward achieving social equity.

Most older adults maintain close social connections, though their network structure evolves as they age (Cornwell et al., 2008). Research generally suggests that while friendship networks shrink into adulthood, network size among family ties such as spouses, siblings, parents, and children tends to remain stable (Wrzus et al., 2013). Cultural beliefs and institutional practices treat these aggregate patterns as universal experiences. For instance, in the United States, default legal rules privilege relatives defined by blood, marriage, or adoption over chosen family (Knauer, 2016), and long-term care policies and public opinion continue to perpetuate the notion that these same institutionally legible family ties, particularly those that are intergenerational, carry the assumed social responsibility for later-life caregiving, even amid diversifying family structures (Wu et al., 2024).

The maintenance of these beliefs and policies reflects heteronormativity, which assumes a linear progression from marriage to childbearing and positions the heterosexual nuclear family as the standard form of social organization (Allen & Mendez, 2018; Lavender-Stott, 2023; Oswald et al., 2005). Queering social connectivity exposes the disconnect between these normative assumptions and the lived realities of those who fall outside them (Allen, 2022; Fish & Russell, 2018). This tension is especially salient for older adults in same-sex couples who, as sexual minorities, have often navigated marginalization from family of origin, cultivated chosen networks outside the bounds of institutional legibility, and grown older outside the heteronormative scripts that structure social life for many older adults (Allen & Lavender-Stott, 2020; Miller, 2023). While recent research has examined the social networks of LGBTQ+ older adults broadly (e.g., Prasad et al., 2025, 2026) and how network configurations differ between lesbian, gay, and bisexual older adults and their heterosexual counterparts (e.g., Hsieh & Liu, 2021; Hsieh & Wong, 2020), scholars have rarely investigated the sex composition of a coresidential partnership as a distinct axis of inquiry. Consequently, it remains unclear whether and how close social networks differ between older adults in same-sex and different-sex unions.

This limitation is notable as older adults in same-sex couples occupy a distinct relational context. Their social networks may differ not only from those in different-sex couples, but also from LGBTQ+ older adults more broadly, for two reasons. First, selection processes may play a role. Most individuals in same-sex coresidential unions identify as gay or lesbian (Julian et al., 2025), and those who enter same-sex coresidential partnerships may be more socially resourced and socioeconomically advantaged than their unpartnered gay and lesbian peers (Carpenter & Gates, 2008; Goldsen et al., 2017; Heaphy, 2009). Second, cohabitation itself may shape network structure independently of selection. Partners are frequently positioned at the core of one’s network (Sun & Schafer, 2023). Living together reinforces this by centering daily routines and social life around the couple, drawing partners inward regardless of couple sex composition (Carrington, 1999). For sexual minorities, coresidential partnership may also confer greater social legibility than remaining unpartnered or in a non-normative arrangement—a dynamic that aligns with homonormativity, in which social acceptance is conditioned on conformity to coupled, stable, and domestic lifestyles (Allen & Mendez, 2018; Duggan, 2002).

Although coresidential partners are a common source of connection in later life (Carr & Utz, 2020), the close ties individuals maintain beyond their partnership remain vital, both as a complement to partnership-based support as couples age together and as a foundation for support in the event of partnership dissolution (Sun & Schafer, 2023). Examining how the composition and size of these networks differ between older adults in same-sex and different-sex couples illuminates the broader relational worlds partnered individuals cultivate alongside their union. Drawing on pooled, probability-based cross-sectional data from the 2006 to 2022 Health and Retirement Study (HRS), this study maps differences in social network composition and size between older adults in same-sex and different-sex couples in the United States. The HRS asks respondents to report the number of close children, immediate family members, and friends in their lives (Smith et al., 2023), enabling an assessment of how the relational form and breadth of close ties vary across partnership sex composition.

## The salience of social network structure

Social relationships are a central determinant of health and well-being in later life (Carr & Utz, 2020). To conceptualize these dynamics, the Convoy Model depicts social connections as concentric circles expanding outward from the individual, with relationships varying in closeness, function, quality, and structure (Antonucci et al., 2014). The present study focuses on the innermost of these circles: the ties that partnered individuals designate as close. The Convoy Model also provides a useful lens for examining variations across diverse family forms (Fuller et al., 2020). Specifically, the model posits that network structures are shaped by personal characteristics, such as sexuality, as well as situational factors, including the historical and cultural contexts of relationship formation (Antonucci et al., 2014), an insight that Tester and Wright (2017) apply to the social convoys of older gay men. The model assumes no built-in deficit for networks that diverge from normative expectations, for instance, allowing friends to occupy the same core structural positions that immediate family or children might otherwise fill (Fuller et al., 2020).

Prior research drawing on the Convoy Model underscores how social network structure shapes the support and resources available in later life, including among LGBTQ+ older adults (e.g., Prasad et al., 2025, 2026). This study examines two structural dimensions: close network size and composition, which reflect how many ties an individual maintains and how those relationships are socially defined. These dimensions are particularly consequential when comparing older adults in same-sex versus different-sex couples, as both the volume of available ties and their relationship category have distinct social implications. A larger network provides broader reserves when partners navigate aging-related challenges or experience relationship dissolution (Sun & Schafer, 2023). This capacity is especially salient for LGBTQ+ older adults, among whom partners perform a disproportionate share of caregiving compared to the broader aging population, where children typically anchor caregiving instead (Knauer, 2016).

Network composition matters for two related reasons. First, “friends” and “family” ties operate under distinct cultural expectations for providing support (Fiori et al., 2020), categories that are themselves socially constructed rather than natural or fixed (Fish et al., 2024). Here, “family” is often denoted to mean ties recognized through blood, marriage, or adoption, a boundary that often excludes chosen family ties that may not be biologically or legally related (Weston, 1991). Intergenerational family ties, such as children or grandchildren, are culturally expected to supply practical assistance, whereas friends are expected to offer emotional support, though these expectations derive primarily from cisgender, heterosexual experiences (Fiori et al., 2020). Second, despite diversifying family structures, U.S. cultural expectations and long-term care policy remain deeply reliant on intergenerational family ties for caregiving in particular (Wu et al., 2024), while default legal frameworks governing surrogate decision-making, guardianship, and inheritance systematically privilege blood, marriage, or adoptive ties over chosen ones (Knauer, 2016). Altogether, relational status dictates both the types of support culturally expected of a close tie and the institutional legibility granted to it.

## Close network composition

Understanding how close social networks vary between older adults in same-sex and different-sex couples requires situating this population within the well-established body of research on LGBTQ+ social networks, which has long documented distinct patterns of network cultivation (e.g., Grossman et al., 2000). In the field-defining ethnography *Families We Choose*, Weston (1991) documented how LGBTQ+ adults, facing marginalization from families of origin, forged alternative networks centered on close friendship and voluntary mutual care. Contemporary research demonstrates that these families of choice remain a defining feature of LGBTQ+ social life today (Hull & Ortyl, 2019).

The Convoy Model theorizes that social networks accumulate across the lifespan rather than forming instantaneously, carrying the imprint of the historical conditions under which they were cultivated (Antonucci et al., 2014). For the current cohort of older adults in same-sex couples, this history includes the stigmatization of same-sex relationships, the AIDS crisis, and systemic exclusion from legal recognition (Knauer, 2016). These distinct life-course experiences explain why friendship ties remain central among LGBTQ+ older adults even amid more recent positive sociopolitical changes (de Vries et al., 2019).

Socioemotional Selectivity Theory (SST) also provides a rationale for why friendship ties are expected to be so heavily concentrated within the “close” layer of the social network. The theory conceptualizes older age as a stage characterized by a strong preference for emotionally meaningful and rewarding connections (Carstensen et al., 1999; English & Carstensen, 2014). As sexual minorities, older adults in same-sex relationships have frequently navigated strained relationships with family of origin, and as such, friendship ties, rooted in voluntary, identity-affirming commitment, were formed as means of emotional safety and mutual validation (Weston, 1991). Consequently, the theorized prioritization of emotionality central to SST helps explain why friends, rather than immediate family, are likely to constitute the majority of non-partner ties designated as “close.”

In constructing chosen families, LGBTQ+ older adults have pioneered queering dominant kinship structures by rejecting the assumption that family is defined through biology or law (Allen & Lavender-Stott, 2020). Nearly two-thirds of LGBT baby boomers report having a family of choice (MetLife Mature Market Institute & Lesbian and Gay Aging Issues Network of the American Society on Aging, 2010), and data from the National Health, Aging, and Sexuality/Gender Study demonstrate that friend-centered networks, frequently in the absence of children, are common among LGBTQ+ older adults (Kim et al., 2017). As such, friendships serve as vital sources of both emotional and practical support within these networks (Croghan et al., 2014; Fredriksen-Goldsen et al., 2011).

At the same time, the social worlds of LGBTQ+ older adults are not reducible to friendship ties alone. Family-of-origin relationships are often complex and multidimensional; qualitative research, for instance, shows that relationships with parents can be characterized by simultaneous solidarity and rejection (Reczek, 2014). Furthermore, some LGBTQ+ older adults occupy roles as parents and grandparents, though less commonly than their cisgender heterosexual peers (Orel & Fruhauf, 2006). For many, chosen family ties complement rather than entirely displace family-of-origin ties (Hull & Ortyl, 2019), and the capacity to cultivate a network composed entirely of chosen ties is itself unevenly distributed, shaped by broader socioeconomic inequalities (Heaphy, 2009).

Despite these rich conceptual foundations, probability-based estimates of network patterns among LGBTQ+ older adults in the U.S. remain limited, as most research has relied on non-probability samples. A notable exception is research by Hsieh and Wong (2020) using the National Social Life, Health, and Aging Project (NSHAP), which demonstrated that LGB older adults report a higher proportion of friends and a lower likelihood of including a partner among their core discussants compared to heterosexual peers. The present study builds on these findings while differing in both measurement strategy and population scope. First, whereas NSHAP assesses core discussant networks by asking respondents to name up to five individuals with whom they discuss important matters, this study uses HRS measures that capture broader counts of close network members across specific relational categories, including children, immediate family, and friends (Smith et al., 2023).

Second, while Hsieh and Wong evaluated LGB older adults across all partnership statuses, this study focuses on partnered older adults in coresidential unions. As mentioned, older adults in same-sex couples represent a distinct subpopulation shaped by selection into partnership (Carpenter & Gates, 2008; Goldsen et al., 2017; Heaphy, 2009), couple-centered domestic routines (Carrington, 1999), and the homonormative legibility of cohabitation (Allen & Mendez, 2018; Duggan, 2002). I hypothesize that older adults in same-sex unions will maintain a higher proportion of friends and a lower proportion of children and immediate family in their close social networks on average than those in different-sex unions, while recognizing that these estimates may diverge from broader LGBTQ+ comparisons due to these specific partner-level processes.

## Close social network size

Whether these hypothesized compositional differences in close network ties extend to differences in network size remains an open question. Hsieh and Wong (2020) provide the only U.S. probability-based estimates that directly address network size, though as mentioned, their comparison evaluated LGB and heterosexual older adults and used a measure that captures a specific subset of connections, confidants turned to for important discussions, and imposes a cap. Drawing on NSHAP data, they found no statistically significant difference in the total number of core discussant ties reported by LGB and heterosexual older adults.

A higher proportion of friendship ties within the close network may affect overall network size because of cohort-specific vulnerabilities. Friendships develop largely among age peers, a pattern driven by age segregation and ageism in social settings (de Vries et al., 2019; Heaphy, 2009). This age homogeneity leaves friend-centered networks vulnerable to simultaneous, cohort-wide exposure to health declines, functional limitations, and mortality in later life (Almack et al., 2010) that may constrain their ability to be closely maintained. For the current cohort of older adults in same-sex couples, these age-related vulnerabilities are compounded by historical losses from the AIDS crisis. The epidemic’s severe mortality burden fell heavily on gay men and their close ties (Hamel et al., 2014), but also impacted the social networks of lesbians, who, in addition to being underreported victims of the epidemic, often served as caregivers and advocates of gay and bisexual men (Faderman, 1991). Coupled with the limited availability of affirming LGBTQ+ “third places” (Boggs et al., 2017), especially given the decline of LGBTQ+ bars (Mattson, 2019), friend-centered networks may face ongoing constraints on tie replacement. Meanwhile, older adults in different-sex couples are more likely to have children and grandchildren, which structurally diversifies their networks by age and may offset constraints on network size in age-peer networks.

An alternative theoretical perspective suggests that the historical trajectory of LGBTQ+ social connectivity is fundamentally one of resilience and adaptive network cultivation (Allen & Lavender-Stott, 2020). For instance, while the AIDS epidemic imposed profound mortality losses, it also forged an enduring culture of mutual caregiving within LGBTQ+ communities (Muraco & Fredriksen-Goldsen, 2011). Specifically, mutual caregiving among friends serves as a key mechanism for fulfilling relational roles, turning close friends into active support systems that sustain closeness despite potentially shared functional constraints (Muraco & Fredriksen-Goldsen, 2011). Moreover, given the strong emotional history of these ties, Socioemotional Selectivity Theory (Carstensen et al., 1999; English & Carstensen, 2014) suggests that older adults in same-sex couples may be especially motivated to maintain them despite aging-related constraints. Furthermore, when ties can no longer be sustained due to mortality, network evolution does not necessarily cease. The Convoy Model conceptualizes social networks as dynamic, lifespan structures that adapt to loss (Antonucci et al., 2014). As such, older adults in same-sex couples may prioritize cultivating ties that fill gaps.

Although cross-sectional data cannot directly capture these underlying processes, these competing perspectives offer distinct theoretical frameworks for interpreting overall network size. Cohort-specific vulnerabilities in friendship ties suggest a rationale for smaller close networks among older adults in same-sex couples, whereas adaptive caregiving norms and selective tie replacement explain how intentional social network maintenance might preserve parity with those in different-sex couples. Because these underlying processes remain unobserved, I treat the direction of potential differences in close network size as an open, exploratory hypothesis.

## Data and methods

This study drew on the HRS Psychosocial and Lifestyle Leave Behind Questionnaire (LBQ; Smith et al., 2023), covering the period from 2006 to 2022. The HRS is a biennial, nationally representative survey of noninstitutionalized adults aged 51 and older, sponsored by the National Institute on Aging and conducted by the University of Michigan. To preserve representativeness, new adult samples are added every 6 years. The LBQ is administered to half of the sample every wave, starting in 2006 (Smith et al., 2023), with response rates ranging from 61.8% to 87.7% (Smith et al., 2023). To maximize the sample of older adults in same-sex couples available for cross-sectional analysis, data were pooled across waves from 2006 to 2022. Variables were drawn from the RAND Core HRS Fat Files (RAND HRS, 2024), the RAND Longitudinal File (RAND HRS, 2024), and the University of Michigan’s HRS Tracker File (Health and Retirement Study, 2024).

The analytic sample was constructed through a series of restrictions applied to the 59,338 person-observations available in the LBQ across this period. First, observations without valid weights were excluded, leaving 55,256 observations. The sample was then restricted to those who were cohabiting or married and observed in a same-sex or different-sex coresidential relationship, meaning neither the respondent’s nor their partner’s sex was missing, yielding 34,797 observations. Next, observations with missing responses to the social network questions were excluded, leaving 29,342 person-observations. Finally, to support cross-sectional estimation rather than change over time, only each respondent’s first-wave observation that met the sample criteria was retained. The final analytic sample includes 134 older adults in same-sex relationships and 12,795 in different-sex relationships.

### Measures

#### Social network structure

The LBQ comprises a series of questions assessing older adults’ social network structure, including the composition and size of their close relationships (Smith et al., 2023). Respondents were asked whether they had any of four relationship types in their social network: spouse or partner, living children, immediate family (“for example, brothers or sisters, parents, cousins, or grandchildren;” Smith et al., 2023), and friends. Because the current study focuses exclusively on older adults in coresidential same-sex and different-sex relationships, the spouse or partner category was excluded, and analyses centered on the remaining three relationship types. Respondents then reported the number of close relationships within each type. To limit the influence of outliers, responses were top-coded at the 99th percentile. Close network size was calculated by summing the number of close relationships across the three relationship types. Network composition was calculated by dividing the number of close relationships in each type by the total network size, yielding the proportion of the overall close network contributed by each relationship type.

#### Sociodemographic characteristics

Several sociodemographic characteristics are included as covariates to account for potential confounders of the relationship between partnership sex composition and network structure. The sex composition of the respondent’s relationship was coded as same-sex partnership (1) or different-sex partnership (0), based on the respondent’s reported sex and that of their partner. Respondent sex was coded as male (1) or female (0). Relationship type distinguished married (1) from cohabiting (0) respondents. Age was treated as a continuous variable. Race and ethnicity were coded as non-Hispanic White (1) versus Hispanic or non-White (0); this binary coding reflects data constraints rather than theoretical assumptions about racial and ethnic heterogeneity. Education was coded as bachelor’s degree or higher (1) versus high school or less (0). Household income was operationalized as the log-transformed sum of the respondent’s and their partner’s income from the previous calendar year, adjusted to 2022 dollars prior to transformation. Employment status distinguished those currently employed (1) from those not employed (0). Survey wave was included as a categorical variable corresponding to interview year, ranging from 2006 to 2022 in 2-year increments.

### Analytic strategy

The analyses began with descriptive statistics for older adults, stratified by coresidential union status (same-sex versus different-sex unions; Table 1). These descriptive statistics summarized key network characteristics, including the presence, count, and proportion of children, immediate family members, and friends, as well as overall network size and sociodemographic characteristics. Next, I evaluated differences in social network structure by the sex composition of relationships, adjusting for sociodemographic covariates. To analyze network composition, I estimated fractional probit models that predict the proportion of close ties that are children (Table 2), immediate family members (Table 3), and friends (Table 4), an approach specifically designed for proportional outcomes bounded between zero and one (Papke & Wooldridge, 1996). Finally, to examine overall network size, I fitted a negative binomial regression model (Table 5) to account for overdispersion inherent in count-based network size data.

**Table 1:** Weighted characteristics of older adults in same-sex and different-sex relationships.

Characteristics	Same-sex	Different-sex	*p*
	Mean/proportion [90% CI]	Mean/proportion [90% CI]	
**Number in sample**	134	13,910	
**Weighted proportion**	0.01 [0.01, 0.01]	0.99 [0.99, 0.99]	
**Social network structure**			
Any children	0.40 [0.30, 0.51]	0.91 [0.90, 0.92]	<.001***
Any immediate family	0.96 [0.88, 0.98]	0.99 [0.99, 0.99]	.21
Any friends	0.95 [0.90, 0.97]	0.91 [0.91, 0.92]	.13
Number of close children	0.84 [0.53, 1.15]	2.12 [2.09, 2.16]	<.001***
Number of close immediate family	1.56 [0.76, 2.36]	2.98 [2.90, 3.06]	.01***
Number of close friends	5.05 [4.16, 5.93]	3.66 [3.59, 3.74]	.01**
Overall close network size	7.45 [5.97, 8.93]	8.77 [8.62, 9.12]	.14
Proportion of children in close network	0.09 [0.07, 0.11]	0.28 [0.27, 0.28]	<.001***
Proportion of immediate family in close network	0.14 [0.09, 0.19]	0.30 [0.30, 0.31]	<.001***
Proportion of friends in close network	0.77 [0.71, 0.83]	0.41 [0.41, 0.42]	<.001***
**Sociodemographic characteristics**			
Male	0.53 [0.42, 0.64]	0.54 [0.53, 0.54]	.94
Married	0.35 [0.26, 0.45]	0.93 [0.91, 0.94]	<.001***
Age	59.01 [57.56, 60.46]	61.45 [61.18, 61.71]	.01**
Non-Hispanic white	0.78 [0.72, 0.83]	0.77 [0.75, 0.78]	.69
Bachelor’s or higher	0.74 [0.62, 0.83]	0.57 [0.55, 0.59]	.01**
Household income (in U.S. dollars)	164,532 [112,762, 216,302]	109,884 [103,096, 116,672]	.09^†^
Employed	0.69 [0.60, 0.77]	0.51 [0.50, 0.52]	.01**

*Note*. CI = confidence interval. Estimates account for the complex survey design. Design-based standard errors were estimated using Taylor series linearization.

Data source: Health and Retirement Study, 2006–2022.

† *p* < .10.

* *p* < .05.

** *p* < .01.

*** *p* < .001.

**Table 2:** Weighted fractional probit regression model predicting the proportion of close children in the network.

Variables	Coefficient (*SE*)	[90% CI]	*p*
**Sex composition of relationship (Ref = Different-sex)**			
Same-sex	−0.67 (0.07)	[−0.79, −0.54]	<.001***
**Sex (Ref = Female)**			
Male	0.06 (0.01)	[0.04, 0.08]	<.001***
**Union-type (Ref = Married)**			
Cohabiting	−0.17 (0.03)	[−0.22, −0.12]	<.001***
**Age**	0.00 (0.00)	[0.00, 0.00]	.01**
**Race/ethnicity (Ref = Hispanic or non-White)**			
Non-Hispanic White	−0.02 (0.02)	[−0.05, 0.00]	.20
**Education**			
Bachelor’s degree or higher	−0.06 (0.01)	[−0.08, −0.04]	<.001***
**Logged household income**	0.00 (0.02)	[−0.01, 0.01]	.68
**Employed (Ref = Not employed)**			
Employed	0.00 (0.02)	[−0.03, 0.03]	.99
**Constant**	−0.74 (0.10)	[−0.90, −0.57]	<.001***

*Note*. *SE* = standard error; CI = confidence interval; Ref = reference. Estimates account for the complex survey design. Design-based standard errors were estimated using Taylor series linearization. The model includes a categorical covariate for the survey wave.

Data source: Health and Retirement Study, 2006–2022.

† *p* < .10.

* *p* < .05.

** *p* < .01.

*** *p* < .001.

**Table 3:** Weighted fractional probit regression model predicting proportion of close immediate family in the network.

Variables	Coefficient (*SE*)	[90% CI]	*p*
**Sex composition of relationship (Ref = Different-sex)**			
Same-sex	−0.54 (0.13)	[−0.76, −0.33]	<.001***
**Sex (Ref = Female)**			
Male	−0.08 (0.01)	[−0.10, −0.05]	<.001***
**Union-type (Ref = Married)**			
Cohabiting	−0.08 (0.03)	[−0.12, −0.03]	.01**
**Age**	0.00 (0.00)	[−0.00, 0.00]	.55
**Race/ethnicity (Ref = Hispanic or non-White)**			
Non-Hispanic White	−0.18 (0.02)	[−0.21, −0.15]	<.001***
**Education**			
Bachelor’s degree or higher	−0.11 (0.02)	[−0.14, −0.09]	<.001***
**Logged Household Income**	−0.01 (0.01)	[−0.02, −0.00]	.09^†^
**Employed (Ref = Not employed)**			
Employed	0.03 (0.02)	[0.00, 0.06]	.08^†^
**Constant**	−0.19 (0.08)	[−0.32, −0.06]	.02**

*Note*. *SE* = standard error; CI = confidence interval; Ref = reference. Estimates account for the complex survey design. Design-based standard errors were estimated using Taylor series linearization. The model includes a categorical covariate for the survey wave.

Data source: Health and Retirement Study, 2006–2022.

† *p* < .10.

* *p* < .05.

** *p* < .01.

*** *p* < .001.

**Table 4:** Weighted fractional probit regression model predicting proportion of friends in the network.

Variables	Coefficient (*SE*)	[90% CI]	*p*
**Sex composition of relationship (Ref = Different-Sex)**			
Same-Sex	0.87 (0.12)	[0.67, 1.07]	<.001***
**Sex (Ref = Female)**			
Male	−0.00 (0.01)	[−0.02, 0.02]	.97
**Union-type (Ref = Married)**			
Cohabiting	0.18 (0.04)	[0.12, 0.24]	<.001***
**Age**	−0.00 (0.00)	[−0.00, −0.00]	.04**
**Race/Ethnicity (Ref = Hispanic or non-White)**			
Non-Hispanic White	0.19 (0.02)	[0.16, 0.22]	<.001***
**Education**			
Bachelor’s degree or higher	0.16 (0.02)	[0.13, 0.19]	<.001***
**Logged household income**	0.01 (0.01)	[−0.00, 0.02]	.26
**Employed (Ref = Not employed)**			
Employed	−0.02 (0.02)	[−0.06, 0.01]	.24
**Constant**	−0.44 (0.12)	[−0.63, −0.24]	<.001***

*Note*. *SE* = standard error; CI = confidence interval. Estimates account for the complex survey design. Design-based standard errors were estimated using Taylor series linearization. The model includes a categorical covariate for the survey wave.

Data source: Health and Retirement Study, 2006–2022.

† *p* < .10.

* *p* < .05.

** *p* < .01.

*** *p* < .001.

**Table 5:** Weighted negative binomial regression model predicting social network size.

Variables	Coefficient (*SE*)	[90% CI]	*p*
**Sex composition of relationship (Ref = Different-sex)**			
Same-sex	−0.00 (0.12)	[−0.21, 0.21]	.99
**Sex (Ref = Female)**			
Male	−0.08 (0.01)	[−0.11, −0.06]	<.001***
**Union-type (Ref = Married)**			
Cohabiting	−0.20 (0.03)	[−0.26, −0.15]	<.001***
**Age**	0.01 (0.00)	[0.01, 0.01]	<.001***
**Race/ethnicity (Ref = Hispanic or non-White)**			
Non-Hispanic White	−0.14 (0.02)	[−0.17, −0.10]	<.001***
**Education**			
Bachelor’s degree or higher	−0.06 (0.02)	[−0.09, −0.03]	<.001***
**Logged Household Income**	−0.00 (0.01)	[−0.02, 0.01]	.81
**Employed (Ref = Not employed)**			
Employed	0.05 (0.02)	[0.01, 0.09]	.04**
**Constant**	1.56 (0.14)	[1.33, 1.79]	<.001***

*Note*. *SE* = standard error; CI = confidence interval; Ref = reference. Estimates account for the complex survey design. Design-based standard errors were estimated using Taylor series linearization. The model includes a categorical covariate for the survey wave.

Data source: Health and Retirement Study, 2006–2022.

† *p* < .10.

* *p* < .05.

** *p* < .01.

*** *p* < .001.

Missing data on covariates were minimal and addressed using imputation with the subgroup mean or mode, stratified by same-sex versus different-sex union status (Groves et al., 2009). To assess the sensitivity of this approach, I also estimated all models using listwise deletion, which yielded substantively similar results. All analyses incorporated wave-specific LBQ sampling weights, along with stratum and half-stratum indicators, to account for the complex survey design of the HRS (Heeringa et al., 2017). Analyses were conducted in Stata 18 using the “svy” suite with the “subpop” specification, and standard errors were estimated using Taylor series linearization. Results are presented with 90% confidence intervals, with statistical significance assessed at an alpha level of 0.10.

## Results

Table 1 presents descriptive characteristics of older adults in same-sex and different-sex unions. With respect to network structure, a substantially smaller proportion of older adults in same-sex unions reported having any children (0.40 [90% CI: 0.30, 0.51]) compared with those in different-sex unions (0.91 [90% CI: 0.90, 0.92]), consistent with the documented lower prevalence of parenthood among LGBTQ+ populations (Wilson & Bouton, 2024). Notably, nearly all older adults in different-sex couples have at least one child, whereas only two in five of those in same-sex couples do. In contrast, the proportions reporting any immediate family members or any friends were remarkably similar.

Turning to close network composition, children constituted a much smaller average share of the close social network among older adults in same-sex unions (0.09 [90% CI: 0.07, 0.11]) than among those in different-sex unions (0.28 [90% CI: 0.27, 0.28]). A similar pattern emerged for immediate family members, who accounted for an average of 0.14 [90% CI: 0.09, 0.19] of close ties among older adults in same-sex unions compared with 0.30 [90% CI: 0.30, 0.31] among those in different-sex unions. Both patterns are consistent with expectations. Conversely, close friends comprised a substantially larger average share of the social networks of older adults in same-sex unions (0.77 [90% CI: 0.71, 0.83]) than among those in different-sex unions (0.41 [90% CI: 0.41, 0.42]). On average, then, close friends accounted for most reported close ties among older adults in same-sex couples, compared with less than half among those in different-sex couples.

Overall, the size of the close network did not differ between the two groups, although it approached marginal significance (*p *= .14), suggesting equivalent social breadth achieved through different relational configurations. Specifically, older adults in same-sex relationships reported fewer close children on average (0.84 [90% CI: 0.53, 1.15] vs 2.12 [90% CI: 2.09, 2.16]), which is attributable to their lower likelihood of reporting any living children. They also reported fewer close immediate family members (1.56 [90% CI: 0.76, 2.36] vs 2.98 [90% CI: 2.90, 3.06]), but more close friends (5.05 [90% CI: 4.16, 5.93] vs 3.66 [90% CI: 3.59, 3.74]).

Older adults in same-sex and different-sex unions also differed across several sociodemographic characteristics. While the proportion reported as male was comparable, a smaller proportion of older adults in same-sex unions were married (0.35 [90% CI: 0.26, 0.45]) than those in different-sex unions (0.93 [90% CI: 0.91, 0.94]). On average, older adults in same-sex unions were younger (59.01 [90% CI: 57.56, 60.46]) than those in different-sex unions (61.45 [90% CI: 61.18, 61.71]). The racial and ethnic composition was similar across groups. Older adults in same-sex unions were more likely to have attained a bachelor’s degree or higher (0.74 [90% CI: 0.62, 0.83]) than those in different-sex unions (0.57 [90% CI: 0.55, 0.59]), and mean household income was correspondingly higher among older adults in same-sex unions ($164,532 [90% CI: $112,762, $216,302]) than among those in different-sex unions ($109,884 [90% CI: $103,096, $116,672]). A higher proportion of older adults in same-sex unions were also employed (0.69 [90% CI: 0.60, 0.77]) than those in different-sex unions (0.51 [90% CI: 0.50, 0.52]). These demographic differences are broadly consistent with estimates from the American Community Survey (Manning & Brown, 2015).

Turning to the multivariable analyses, results from the weighted fractional probit models revealed marked differences in network composition, even after adjusting for sociodemographic characteristics. As shown in Table 2, older adults in same-sex unions had a significantly smaller proportion of close children in their social networks than those in different-sex unions (*β* = −0.67, *p *< .001). Table 3 similarly indicates a smaller proportion of close immediate family members among the network of older adults in same-sex unions (*β* = −0.54, *p *< .001). Conversely, Table 4 shows that older adults in same-sex unions reported a significantly higher proportion of close friends (*β* = 0.87, *p *< .001). Finally, Table 5 presents estimates from the weighted negative binomial regression model predicting close network size. Consistent with the descriptive findings, this model indicates no significant difference in overall network size between older adults in same-sex and different-sex unions (*β* = −0.00, *p *= .99).

### Sensitivity checks

Although the HRS provides a rare opportunity to examine older adults in same-sex and different-sex unions using probability-based data, the relatively small number of respondents in same-sex partnerships reflects the absence of oversampling strategies targeting LGBTQ+ individuals or same-sex couples, which increases the risk of Type II error and limits analyses of within-group heterogeneity (Liu et al., 2021). Within-group heterogeneity is theoretically important because sexual identity does not operate in isolation but intersects with sex/gender and race to shape exposure to marginalization and access to social resources across the life course (Allen & Mendez, 2018), which may matter for social network cultivation. Prior research also documents meaningful variation within same-sex couples by union status, sex, and race and ethnicity in the prevalence of parenthood (Wilson & Bouton, 2024), and parenthood appears to account for at least part of the compositional differences observed in the current study.

Supplemental unadjusted analyses examined whether the aggregate patterns varied by sex, union status, and race and ethnicity. In these analyses, first differences capture the same-sex versus different-sex gap within a given subgroup, while second differences test whether that gap itself varies significantly across subgroups. Although these estimates should be interpreted with caution, given the small cell sizes, they provide suggestive evidence of heterogeneity. With respect to network composition (Supplementary Tables 1–3), the differences in the proportions of friends and children between older adults in same-sex and different-sex unions were more pronounced among males than among females. Similarly, the difference in the proportion of friends between older adults in same-sex and different-sex couples was larger among those who were cohabiting than among those who were married. With respect to network size (Supplementary Table 4), the average network size of females in same-sex and different-sex couples was equivalent; however, males in same-sex couples reported significantly smaller close networks than males in different-sex couples, with a difference of approximately 3.08 fewer ties [90% CI: −4.24, −1.91] on average. None of the second differences for race/ethnicity were statistically significant at the 0.10 alpha level. Supplementary Tables 5–8 provide estimates adjusting for sociodemographic characteristics and reveal the same underlying pattern.

## Discussion

Using probability-based data from the HRS, this study examined differences in the composition and size of close social networks among partnered older adults. The findings show that older adults in same-sex couples maintain a distinctly friend-centered close network beyond their partner, reporting a significantly higher proportion of friends and a lower proportion of immediate family and children among the ties they designate as close. Indeed, while friendships accounted for less than half of these close non-partner ties on average among older adults in different-sex couples, friends constituted the clear majority of close ties for those in same-sex unions. This contrast shows that partnership sex composition is associated with who occupies close relational positions beyond a partner.

Heteronormativity presumes marriage and childbearing as the default lifespan trajectory, establishing the heterosexual nuclear family as the normative framework of social organization (Allen & Mendez, 2018; Lavender-Stott, 2023; Oswald et al., 2005). Within the Convoy Model, a central premise is that friends can occupy the same core structural positions as immediate family or children (Fuller et al., 2020), and these findings directly support that notion. As shown in the descriptive findings, while the prevalence of living children differed, nearly all older adults in both same-sex and different-sex partnerships reported having immediate family and friends. However, what differed was the designation of these ties as close. Friendships more often carry that designation than immediate family for those in same-sex couples, because friend networks have historically functioned as primary sites of mutual aid, emotional intimacy, and identity affirmation for LGBTQ+ populations amid marginalization from their family of origin (Weston, 1991). Consequently, because older adults often prioritize relationships that feel emotionally meaningful and rewarding (Carstensen et al., 1999; English & Carstensen, 2014), friendship ties are likely to meet the threshold for “close” in ways that immediate family ties do not.

In contrast to these compositional differences, the results demonstrate no significant difference in overall close network size, as partnered older adults in same-sex and different-sex couples reported similar numbers of close non-partner ties. Because friendships are largely horizontal (age-peer) rather than vertical (intergenerational), friend-reliant networks are presumed to be more vulnerable to late-life peer illness and mortality than children-anchored networks (Almack et al., 2010), which may, in turn, constrain their ability to be “closely” maintained. While friend-centered convoys carry distinct age-graded vulnerabilities, a predominantly friend-based close network did not correspond to a smaller overall network size in this study. Although cross-sectional data cannot directly capture dynamic network churn or tie substitution over time, this parity aligns with frameworks of LGBTQ+ aging that emphasize relational resilience and adaptive kinship across the life course (Allen & Lavender-Stott, 2020).

These findings both align with and extend the limited probability-based literature on LGBTQ+ older adults’ social networks in the US, most notably Hsieh and Wong (2020). Whereas Hsieh and Wong (2020) examined LGB older adults regardless of partnership status using NSHAP core discussion rosters (which cap respondents), the present study focused specifically on partnered older adults in same-sex versus different-sex couples in the HRS, which captures the distinct number of close ties respondents report across three explicit categories: friends, immediate family, and children. Despite these distinct measurement strategies, both studies converge on two key patterns: same-sex partnered and LGB older adults report a significantly higher proportion of friends among their close ties than their different-sex partnered and heterosexual peers, while maintaining a comparable overall number of reported ties.

That this friend-centered composition and volume parity hold across both overall capped confidant rosters (NSHAP) and category-specific closeness counts (HRS) underscores the robustness of friendship in same-sex partnered and LGB late-life social networks. At the same time, focusing on partnered older adults fills a key gap, showing that friend-centered networks remain a defining feature of social connectivity even within coresidential unions. Future probability-based research with larger samples of partnered and unpartnered LGBTQ+ respondents will be essential to test whether partnership status moderates network structure differences between LGBTQ+ older adults and their cis-heterosexual counterparts.

Supplemental analyses, however, suggest that this overall pattern of size parity and friend composition conceals heterogeneity across subgroups. First, the gap in friend composition between older adults in same-sex and different-sex couples was markedly wider among those who were cohabiting than married. This pattern aligns with the notion of homonormativity (Allen & Mendez, 2018; Duggan, 2002), which posits that legal marriage confers a degree of social legibility and, in this case, may facilitate greater integration into a more normative network structure. Patterns also differed between males and females. The gap in the proportions of close friends and children between older adults in same-sex and different-sex couples was significantly larger among males than among females. Similarly, a gap in overall network size emerged exclusively among males, who maintained smaller networks than their different-sex counterparts, with no comparable difference between females in same-sex and different-sex unions. Indeed, partnered males in same-sex couples reported the highest proportion of friend ties and the smallest overall close networks of any group, driven almost entirely by fewer close ties to children and immediate family, which friend ties could not fully offset on average.

Framed through the Convoy Model, this distinct network profile may reflect how socio-historical forces shape social convoys across the life course. Specifically, older gay and bisexual males came of age during an era marked by intense sexual stigma and institutional hostility toward particularly male same-sex relationships relative to female same-sex relationships (Herek, 2002), constraining the maintenance of family of origin ties (Weston, 1991). This structural landscape was further compounded by historical barriers to parenthood, particularly for male-male couples (Baumle & Compton, 2015) and the profound, enduring loss of peers during the AIDS epidemic (Hamel et al., 2014). Together, these subgroup variations show how broad population averages obscure subgroup differences that larger samples should disentangle more thoroughly.

Although this study provides new probability-based insights into the close network structures of partnered older adults, several limitations warrant consideration. First, this analysis measures partnership sex composition rather than individual sexual or gender identity. Sexual diversity and relationship structure are distinct constructs. Many sexually diverse individuals are not in same-sex partnerships, and not all individuals in different-sex couples are cisgender or heterosexual (Julian et al., 2025). While this focus reflects broader historical constraints in probability-based surveys of aging (Flatt et al., 2022), it precludes direct comparisons with unpartnered LGBTQ+ older adults.

Second, the HRS social network measures introduce two related limitations. Tie closeness is treated as binary, and only non-partner ties that respondents explicitly designate as close are recorded. As a result, peripheral, strained, or ambivalent connections are omitted entirely, leaving the full spectrum of relational closeness uncaptured. This omission may be especially consequential for LGBTQ+ older adults as they navigate family of origin ties that resist classification within a simple binary closeness threshold (e.g., Reczek, 2014). Third, the HRS framework also imposes a rigid “friend versus family” categorization that may not reflect how LGBTQ+ communities label their closest ties. Friendship relationships in LGBTQ+ communities can be framed through familial labels, as when a close friend is described as a brother or sister (e.g., Muraco, 2006). Respondents who conceptualize chosen family in these terms may list such ties under immediate family rather than friends. This means the observed proportion of friends in the HRS may underestimate the prevalence of close non-biological or legal ties among older adults in same-sex couples. This categorization challenge points to a broader conceptual issue. Because relationship categories are socially constructed (Fish et al., 2024), analyzing people’s closest ties through their institutional recognition, rather than a friend-versus-family binary, may better capture the relational boundaries that can drive late-life social inequalities.

A similar granularity issue affects the children category. Because the survey asks broadly about children without constraining response options to legal or biological parent–child relationships, as is common in survey measures, it captures ties within LGBTQ+ families that are often missed (Baumle & Compton, 2014). However, this breadth collapses distinct pathways to parenthood into a single category, obscuring the institutional legibility of the parent–child relationships respondents navigate. Because many LGBTQ+ older adults raised children during eras marked by severe legal restrictions on second-parent adoption and parental recognition (Movement Advancement Project, 2023), these unobserved distinctions carry forward into later life, and, for instance, may shape default legal protections between aging parents and their adult children.

Fourth, the HRS partnership measure assumes a mononormative relationship structure by capturing only a single partner, thereby omitting additional partners in multi-partner configurations, which is a limitation that disproportionately affects LGBTQ+ populations, who report higher prevalence of non-monogamous unions (Compton & Kaufman, 2024). In other words, although the current study focuses on non-partner ties, the partnered older adults in this study may have more than one partner, who may also be part of their close network. Finally, due to sample size constraints common in national probability datasets, data were pooled across multiple survey waves spanning periods of sociopolitical change, potentially obscuring meaningful temporal variation.

Even with these limitations, this work advances the study of later-life families by demonstrating that older adults in same-sex couples cultivate distinctly friend-centered close networks beyond their partner. Because partners provide a disproportionate amount of caregiving for LGBTQ+ older adults compared with the broader aging population (Knauer, 2016), non-partner ties may become especially essential for filling support gaps when both partners face aging-related challenges simultaneously, or when the partnership itself dissolves (Sun & Schafer, 2023). Despite the growing diversity of families, U.S. policies surrounding default legal roles and long-term care are still built around the heterosexual nuclear family (Knauer, 2016; Wu et al., 2024). These findings affirm the importance of policies and practices that acknowledge diverse family arrangements, particularly those rooted in chosen kinship, as a fundamental step toward achieving social equity.

## Supplementary Material

gbag185_Supplementary_Data

## Data Availability

Health and Retirement Study (HRS) data are publicly available. This study was not preregistered.
