# Supplementary material for: Composition and size of close social networks among older adults in same-sex and different-sex couples
Source: J Gerontol B Psychol Sci Soc Sci. 2026 Sep 10;81(11):gbag185. doi: 10.1093/geronb/gbag185 (PMC13706616; doi:10.1093/geronb/gbag185)
Supplement: Supplementary file 1 [file gbag185_supplementary_data.docx]

***The Journals of Gerontology, Series B: Psychological Sciences and Social Sciences* Supplementary Material: Julian. Composition and size of close social networks among older adults in same-sex and different-sex couples.**

**Contents**

[**Supplementary Table 1**. Unadjusted proportion of children in network, 90% confidence intervals in brackets	2](#_Toc238510982)

[**Supplementary Table 2**. Unadjusted proportion of immediate family in network, 90% confidence intervals in brackets	3](#_Toc238510983)

[**Supplementary Table 3**. Unadjusted proportion of friends in network, 90% confidence intervals in brackets	4](#_Toc238510984)

[**Supplementary Table 4**. Unadjusted network size, 90% confidence intervals in brackets	5](#_Toc238510985)

[**Supplementary Table 5**. Adjusted proportion of children in network, 90% confidence intervals in brackets	6](#_Toc238510986)

[**Supplementary Table 6**. Adjusted proportion of immediate family in network, 90% confidence intervals in brackets	7](#_Toc238510987)

[**Supplementary Table 7**. Adjusted proportion of friends in network, 90% confidence intervals in brackets	8](#_Toc238510988)

[**Supplementary Table 8**. Adjusted network size, 90% confidence intervals in brackets	9](#_Toc238510989)

# **Supplementary Table 1**. Unadjusted proportion of children in network, 90% confidence intervals in brackets

|  | **Same-Sex** | **Different-Sex** | **First Difference** | **Second Difference** |
| --- | --- | --- | --- | --- |
| Sex |  |  |  |  |
| Male | 0.05  [0.02, 0.08] | 0.29  [0.28, 0.29] | **-0.24**  **[-0.27, -0.20]** | **-0.10**  **[-0.16, -0.04]** |
| Female | 0.13  [0.09, 0.17] | 0.27  [0.26, 0.27] | **-0.14**  **[-0.17, -0.10]** |  |
| Race/Ethnicity |  |  |  |  |
| Non-Hispanic White | 0.08  [0.06, 0.11] | 0.28  [0.27, 0.28] | **-0.19**  **[-0.22, -0.17]** | -0.02  [-0.09, 0.05] |
| Hispanic or non-White | 0.11  [0.05, 0.17] | 0.28  [0.28, 0.29] | **-0.18**  **[-0.24, -0.12]** |  |
| Union-type |  |  |  |  |
| Cohabiting | 0.06  [0.03, 0.09] | 0.23  [0.21, 0.24] | **-0.17**  **[-0.20, -0.14]** | -0.03  [-0.09, 0.04] |
| Married | 0.14  [0.09, 0.18] | 0.28  [0.28, 0.29] | **-0.14**  **[-0.19, -0.10]** |  |

Note: Estimates account for the complex survey design. Design-based standard errors were estimated using Taylor series linearization. First differences refer to comparisons between older adults in same-sex and different-sex couples. Second differences refer to whether the gap between those in same-sex and different-sex couples is equivalent across sex, race/ethnicity, and union-type. Significant differences at the alpha level of 0.10 are bolded.

Data source: Health and Retirement Study, 2006-2022.

# **Supplementary Table 2**. Unadjusted proportion of immediate family in network, 90% confidence intervals in brackets

|  | **Same-Sex** | **Different-Sex** | **First Difference** | **Second Difference** |
| --- | --- | --- | --- | --- |
| Sex |  |  |  |  |
| Male | 0.09  [0.03, 0.15] | 0.29  [0.29, 0.30] | **-0.20**  **[-0.26, -0.14]** | -0.07  [-0.17, 0.02] |
| Female | 0.19  [0.12, 0.26] | 0.32  [0.31, 0.32] | **-0.13**  **[-0.20, -0.06]** |  |
| Race/Ethnicity |  |  |  |  |
| Non-Hispanic White | 0.11  [0.06, 0.15] | 0.29  [0.28, 0.29] | **-0.18**  **[-0.22, -0.13]** | -0.06  [-0.20, 0.09] |
| Hispanic or non-White | 0.23  [0.09, 0.38] | 0.36  [0.35, 0.37] | -0.12  [-0.27, 0.02] |  |
| Union-type |  |  |  |  |
| Cohabiting | 0.10  [0.05, 0.15] | 0.30  [0.28, 0.31] | **-0.20**  **[-0.25, -0.14]** | -0.10  [-0.20, 0.00] |
| Married | 0.21  [0.12, 0.30] | 0.31  [0.30, 0.31] | **-0.10**  **[-0.19, -0.01]** |  |

Note: Estimates account for the complex survey design. Design-based standard errors were estimated using Taylor series linearization. First differences refer to comparisons between older adults in same-sex and different-sex couples. Second differences refer to whether the gap between those in same-sex and different-sex couples is equivalent across sex, race/ethnicity, and union-type. Significant differences at the alpha level of 0.10 are bolded.

Data source: Health and Retirement Study, 2006-2022.

# **Supplementary Table 3**. Unadjusted proportion of friends in network, 90% confidence intervals in brackets

|  | **Same-Sex** | **Different-Sex** | **First Difference** | **Second Difference** |
| --- | --- | --- | --- | --- |
| Sex |  |  |  |  |
| Male | 0.85  [0.77, 0.94] | 0.41  [0.40, 0.42] | **0.44**  **[0.36, 0.53]** | **0.18**  **[0.05, 0.31]** |
| Female | 0.68  [0.59, 0.77] | 0.41  [0.41, 0.42] | **0.27**  **[0.17, 0.36]** |  |
| Race/Ethnicity |  |  |  |  |
| Non-Hispanic White | 0.81  [0.75, 0.86] | 0.43  [0.42, 0.44] | **0.38**  **[0.32, 0.43]** | -0.07  [-0.14, 0.28] |
| Hispanic or non-White | 0.66  [0.46, 0.86] | 0.35  [0.34, 0.36] | **0.31**  **[0.11, 0.51]** |  |
| Union-type |  |  |  |  |
| Cohabiting | 0.84  [0.76, 0.91] | 0.46  [0.44, 0.48] | **0.38**  **[0.30, 0.46]** | **0.13**  **[0.00, 0.27]** |
| Married | 0.65  [0.54, 0.76] | 0.41  [0.40, 0.41] | **0.24**  **[0.13, 0.35]** |  |

Note: Estimates account for the complex survey design. Design-based standard errors were estimated using Taylor series linearization. First differences refer to comparisons between older adults in same-sex and different-sex couples. Second differences refer to whether the gap between those in same-sex and different-sex couples is equivalent across sex, race/ethnicity, and union-type. Significant differences at the alpha level of 0.10 are bolded.

Data source: Health and Retirement Study, 2006-2022.

# **Supplementary Table 4**. Unadjusted network size, 90% confidence intervals in brackets

|  | **Same-Sex** | **Different-Sex** | **First Difference** | **Second Difference** |
| --- | --- | --- | --- | --- |
| Sex |  |  |  |  |
| Male | 5.46  [4.31, 6.61] | 8.53  [8.35, 8.72] | **-3.08**  **[-4.24, -1.91]** | **-3.76**  **[-6.96, -0.56]** |
| Female | 9.72  [6.93, 12.50] | 9.04  [8.87, 9.20] | 0.67  [-2.12, 3.48] |  |
| Race/Ethnicity |  |  |  |  |
| Non-Hispanic White | 6.49  [4.83, 8.15] | 8.54  [8.39, 8.69] | **-2.05**  **[-3.72, -0.38]** | -3.40  [-8.31, 1.50] |
| Hispanic or non-White | 10.85  [6.44, 15.26] | 9.50  [9.23, 9.77] | 1.35  [-3.05, 5.76] |  |
| Union-type |  |  |  |  |
| Cohabiting | 7.18  [5.24, 9.13] | 7.23  [6.85, 7.62] | -0.05  [-2.06, 1.96] | -0.90  [-2.71, 4.50] |
| Married | 7.94  [5.21, 10.67] | 8.88  [7.74, 9.03] | -0.94  [-3.67, 1.78] |  |

Note: Estimates account for the complex survey design. Design-based standard errors were estimated using Taylor series linearization. First differences refer to comparisons between older adults in same-sex and different-sex couples. Second differences refer to whether the gap between those in same-sex and different-sex couples is equivalent across sex, race/ethnicity, and union-type. Significant differences at the alpha level of 0.10 are bolded.

Data source: Health and Retirement Study, 2006-2022.

# **Supplementary Table 5**. Adjusted proportion of children in network, 90% confidence intervals in brackets

|  | **Same-Sex** | **Different-Sex** | **First Difference** | **Second Difference** |
| --- | --- | --- | --- | --- |
| Sex |  |  |  |  |
| Male | 0.07  [0.03, 0.11] | 0.29  [0.28, 0.29] | **-0.22**  **[-0.26, -0.18]** | **-0.09**  **[-0.15, -0.03]** |
| Female | 0.14  [0.10, 0.17] | 0.27  [0.26, 0.27] | **-0.13**  **[-0.16, -0.09]** |  |
| Race/Ethnicity |  |  |  |  |
| Non-Hispanic White | 0.10  [0.07, 0.12] | 0.28  [0.27, 0.28] | **-0.18**  **[-0.20, -0.15]** | -0.02  [-0.08, 0.05] |
| Hispanic or non-White | 0.12  [0.06, 0.18] | 0.28  [0.27, 0.29] | **-0.16**  **[-0.22, -0.10]** |  |
| Union-type |  |  |  |  |
| Cohabiting | 0.06  [0.04, 0.09] | 0.23  [0.21, 0.24] | **-0.17**  **[-0.20, -0.13]** | -0.02  [-0.09, 0.04] |
| Married | 0.14  [0.09, 0.18] | 0.28  [0.28, 0.29] | **-0.14**  **[-0.19, -0.10]** |  |

Note: Estimates account for the complex survey design. Design-based standard errors were estimated using Taylor series linearization. First differences refer to comparisons between older adults in same-sex and different-sex couples. Second differences refer to whether the gap between those in same-sex and different-sex couples is equivalent across sex, race/ethnicity, and union-type. Significant differences at the alpha level of 0.10 are bolded. Estimates are adjusted for the controls used in the main results and calculated using predicted probabilities from fractional probit models that included an interaction term between the sex composition indicator and the variable of interest. Covariates are held at their means.

Data source: Health and Retirement Study, 2006-2022.

# **Supplementary Table 6**. Adjusted proportion of immediate family in network, 90% confidence intervals in brackets

|  | **Same-Sex** | **Different-Sex** | **First Difference** | **Second Difference** |
| --- | --- | --- | --- | --- |
| Sex |  |  |  |  |
| Male | 0.10  [0.04, 0.16] | 0.29  [0.28, 0.30] | **-0.19**  **[-0.26, -0.13]** | -0.07  [-0.16, 0.02] |
| Female | 0.20  [0.13, 0.26] | 0.32  [0.31, 0.32] | **-0.12**  **[-0.19, -0.05]** |  |
| Race/Ethnicity |  |  |  |  |
| Non-Hispanic White | 0.12  [0.07, 0.16] | 0.29  [0.28, 0.29] | **-0.17**  **[-0.22, -0.13]** | -0.06  [-0.21, 0.08] |
| Hispanic or non-White | 0.24  [0.10, 0.38] | 0.35  [0.34, 0.36] | -0.11  [-0.25, 0.03] |  |
| Union-type |  |  |  |  |
| Cohabiting | 0.10  [0.05, 0.15] | 0.28  [0.27, 0.30] | **-0.18**  **[-0.24, -0.13]** | -0.08  [-0.17, 0.01] |
| Married | 0.20  [0.12, 0.28] | 0.31  [0.30, 0.31] | **-0.10**  **[-0.18, -0.02]** |  |

Note: Estimates account for the complex survey design. Design-based standard errors were estimated using Taylor series linearization. First differences refer to comparisons between older adults in same-sex and different-sex couples. Second differences refer to whether the gap between those in same-sex and different-sex couples is equivalent across sex, race/ethnicity, and union-type. Significant differences at the alpha level of 0.10 are bolded. Estimates are adjusted for the controls used in the main results and calculated using predicted probabilities from fractional probit models that included an interaction term between the sex composition indicator and the variable of interest. Covariates are held at their means.

Data source: Health and Retirement Study, 2006-2022.

# **Supplementary Table 7**. Adjusted proportion of friends in network, 90% confidence intervals in brackets

|  | **Same-Sex** | **Different-Sex** | **First Difference** | **Second Difference** |
| --- | --- | --- | --- | --- |
| Sex |  |  |  |  |
| Male | 0.82  [0.72, 0.92] | 0.41  [0.40, 0.42] | **0.41**  **[0.31, 0.51]** | **0.17**  **[0.04, 0.30]** |
| Female | 0.66  [0.57, 0.74] | 0.41  [0.41, 0.42] | **0.24**  **[0.16, 0.33]** |  |
| Race/Ethnicity |  |  |  |  |
| Non-Hispanic White | 0.77  [0.71, 0.83] | 0.43  [0.42, 0.43] | **0.34**  **[0.28, 0.41]** | 0.07  [-0.13, 0.27] |
| Hispanic or non-White | 0.63  [0.44, 0.82] | 0.36  [0.35, 0.36] | **0.27**  **[0.08, 0.46]** |  |
| Union-type |  |  |  |  |
| Cohabiting | 0.83  [0.76, 0.91] | 0.47  [0.45, 0.49] | **0.36**  **[0.28, 0.44]** | **0.12**  **[0.00, 0.24]** |
| Married | 0.65  [0.56, 0.75] | 0.41  [0.40, 0.41] | **0.25**  **[0.15, 0.34]** |  |

Note: Estimates account for the complex survey design. Design-based standard errors were estimated using Taylor series linearization. First differences refer to comparisons between older adults in same-sex and different-sex couples. Second differences refer to whether the gap between those in same-sex and different-sex couples is equivalent across sex, race/ethnicity, and union-type. Significant differences at the alpha level of 0.10 are bolded. Estimates are adjusted for the controls used in the main results and calculated using predicted probabilities from fractional probit models that included an interaction term between the sex composition indicator and the variable of interest. Covariates are held at their means.

Data source: Health and Retirement Study, 2006-2022.

# **Supplementary Table 8**. Adjusted network size, 90% confidence intervals in brackets

|  | **Same-Sex** | **Different-Sex** | **First Difference** | **Second Difference** |
| --- | --- | --- | --- | --- |
| Sex |  |  |  |  |
| Male | 6.53  [5.10, 7.97] | 8.36  [8.20, 8.52] | **-1.82**  **[-3.29, -0.37]** | **-3.96**  **[-7.77, -0.16]** |
| Female | 11.17  [7.82, 14.52] | 9.04  [8.88, 9.19] | 2.13  [-1.23, 5.50] |  |
| Race/Ethnicity |  |  |  |  |
| Non-Hispanic White | 7.63  [5.53, 9.73] | 8.40  [8.26, 8.55] | -0.77  [-2.87, 1.32] | -3.81  [-8.56, 0.94] |
| Hispanic or non-White | 12.63  [8.51, 16.75] | 9.59  [9.32, 9.87] | 3.04  [-1.07, 7.14] |  |
| Union-type |  |  |  |  |
| Cohabiting | 7.51  [5.39, 9.62] | 7.16  [6.78, 7.54] | 0.35  [-1.85, 2.55] | 1.06  [-2.25, 4.36] |
| Married | 8.10  [5.82, 10.38] | 8.81  [8.68, 8.94] | -0.71  [-2.97, 1.56] |  |

Note: Estimates account for the complex survey design. Design-based standard errors were estimated using Taylor series linearization. First differences refer to comparisons between older adults in same-sex and different-sex couples. Second differences refer to whether the gap between those in same-sex and different-sex couples is equivalent across sex, race/ethnicity, and union-type. Significant differences at the alpha level of 0.10 are bolded. Estimates are adjusted for the controls used in the main results and calculated using predicted probabilities from negative binomial models that included an interaction term between the sex composition indicator and the variable of interest. Covariates are held at their means.

Data source: Health and Retirement Study, 2006-2022.
